# T2 Hepatocellular Carcinoma Exception Policies That Prolong Waiting Time Improve the Use of Evidence-based Treatment Practices

**DOI:** 10.1097/TXD.0000000000001039

**Published:** 2020-08-21

**Authors:** Claire Durkin, David E. Kaplan, Therese Bittermann

**Affiliations:** 1 Department of Medicine, University of Pennsylvania, Philadelphia, PA.; 2 Division of Gastroenterology and Hepatology, University of Pennsylvania, Philadelphia, PA.; 3 Division of Gastroenterology, Corporal Michael J. Crescenz VA Medical Center, Philadelphia, PA.; 4 Department of Biostatistics Epidemiology and Informatics, Center for Clinical Epidemiology and Biostatistics, University of Pennsylvania, Philadelphia, PA.

## Abstract

**Background.:**

A United Network for Organ Sharing policy change in 2015 created a 6-mo delay in the receipt of T2 hepatocellular carcinoma exception points. It was hypothesized that the policy changed locoregional therapy (LRT) practices and explant findings because of longer expected waiting time.

**Methods.:**

Patients transplanted with a first T2 hepatocellular carcinoma exception application between January 1, 2010 and December 31, 2014 (prepolicy; N = 6562), and those between August 10, 2015 and December 2, 2019 (postpolicy; N = 2345), were descriptively compared using data from United Network for Organ Sharing.

**Results.:**

Median time from first application to transplantation was more homogenous across the US postpolicy, due to greater absolute increases in Regions 3, 6, 10, and 11 (>120 d). During waitlisting, postpolicy candidates received more LRT overall (*P* < 0.001), with more notable increases in previously short-wait regions. Postpolicy explants were overall more likely to have ≥1 tumor with complete necrosis (23.9 versus 18.4%; *P* < 0.001) and less likely have ≥1 tumor with no necrosis (32.6% versus 38.5%; *P* < 0.001). Significant geographic variability in explant treatment response was observed prepolicy with recipients in previously short-wait regions having more frequent tumor viability at transplant. Postpolicy, there were no differences in the prevalence of recipients with ≥1 tumor with 100% or 0% necrosis across regions (*P* = 0.9 and 0.2, respectively).

**Conclusions.:**

The 2015 T2 exception policy has led to reduced geographic variability in the use of pretransplant LRT and in less frequent tumor viability on explant for recipients in previously short-waiting times.

## Abstract 2

Supplemental Digital Content is available in the text.

## INTRODUCTION

Since 2002, the model for end-stage liver disease (MELD) score, an estimate of the likelihood of death without transplant within 90 d, has been used to assign waitlist priority for liver transplant (LT) candidates. Because MELD scores do not accurately predict mortality for all conditions, transplant centers may apply for exception points to adjust transplant listing priority to better reflect disease severity, allowing for increased equipoise among a heterogeneous patient population.^1–3^

Today, nearly 20%–25% of transplant recipients receive exception points for T2 hepatocellular carcinoma (HCC).^1,4–6^ Although in part due to the increasing prevalence of HCC,^7^ the exception point policy inadvertently led to overprioritization of such patients, relative to non-HCC candidates.^4,6,8,9^ To address this disparity, the United Network for Organ Sharing (UNOS) implemented a policy for patients with HCC on October 8, 2015, that delayed the receipt of MELD exception points by 6 mo. Subsequently, patients were granted 28 exception points with increases every 3 mo up to a maximum score of 34 points.^10–12^ Following the policy change, a modest reduction in the overprioritization of HCC candidates was observed and also a near normalization of waitlist mortality/dropout rate.^13^ Brondfield et al demonstrated that this policy particularly prolonged waiting times in short-wait regions, where the greatest absolute change in dropout was also seen.^14^ None of the studies thus far have demonstrated an effect on posttransplant mortality.^15^

Current treatment guidelines recommend that HCC patients undergo bridging locoregional therapy (LRT) before transplant.^16^ Pretransplant radiologic response and complete pathologic response to pretransplant LRT significantly decrease post-LT HCC recurrence.^17–19^ However, if expected LT waiting time is short, aggressive LRT may be less likely to be pursued. We hypothesized that the 2015 policy resulted in more aggressive pre-LT treatment for HCC, and as a result more prevalent complete pathologic response on LT explant, particularly in regions with shorter prepolicy wait-times.

## MATERIALS AND METHODS

### Data Source, Study Design, and Study Population

This was a retrospective cohort study using the UNOS database.^20^ The prepolicy cohort included all adult waitlisted candidates with a first T2 HCC exception application submitted between December 1, 2010, and December 31, 2014. The postpolicy cohort included all candidates with a first exception application submitted on or after October 8, 2015. December 31, 2014, was chosen as the end-date of the prepolicy period as this ensured that over 97% of candidates were removed from the waitlist either due to transplant or other causes by September 31, 2015, which minimized the proportion of candidates who overlapped with the postpolicy period. The last application in the postpolicy cohort was filed on February 12, 2019.

Subjects were excluded if they (1) were <18 y of age at the time of waitlisting, (2) had undergone prior LT, and (3) were listed for and received a multiorgan transplant. In the analyses focusing on transplanted candidates, the small number prepolicy subjects who underwent LT after September 31, 2015, were excluded (N = 179 of 5844 LTs). In addition, all analyses were restricted to patients who had active T2 HCC exception points at the time of waitlist removal (or at end of follow-up for postpolicy patients (ie, UNOS variable “exc_case” = “Yes”). This exclusion was implemented as tumor characteristics cannot be readily obtained from UNOS data beyond the exception point period.

### Variables

Variables evaluated at waitlisting included as follows: age, gender, race/ethnicity, primary etiology of liver disease, and laboratory MELD score. Laboratory and match MELD were also obtained at LT. Time from waitlisting to first exception application and from first exception application to waitlist removal was measured. Preexception application tumor number, largest tumor size, and LRT received were obtained from the first exception application. Receipt of LRT was evaluated with respect to number and type of treatments used. Ablative therapies included radiofrequency, chemical, thermal ablation, and cryoablation. Receipt of any LRT included all ablative therapies, transarterial chemoembolization, radiation microspheres, or external beam radiation.

The total number of exception applications submitted during waitlisting was obtained. LRT practices were assessed serially throughout the exception waitlist period in candidates with >1 T2 HCC exception application. Among candidates transplanted, tumor number, and largest tumor size were also evaluated from the last exception application submitted before LT. Alpha-fetoprotein (AFP) level at first and last exception application was categorized as ≤20, 21–99, 100–499, and ≥500 ng/mL.^21^ Of note, all data reported from the last exception application was restricted to subjects transplanted with >1 exception application during waitlisting.

Using the UNOS explant file, tumor number and largest tumor size were obtained. Vascular invasion included either microvascular or macrovascular invasion of any tumor reported. Extrahepatic spread included lymph node involvement and distant metastases. The prevalence of subjects with at least 1 tumor with complete necrosis (ie, complete pathologic response) was obtained, as well as the prevalence of subjects with at least 1 tumor with no necrosis (ie, an untreated tumor). Worst tumor differentiation was categorized as well- or moderately differentiated versus poorly differentiated. False-positive HCC was defined as answering “no” to the question “was HCC (viable or nonviable) found in the explant?” as per prior published work.^22^

Variation by UNOS Region with respect to median waiting time from first exception application to LT prepolicy and postpolicy was investigated. Patients in UNOS Regions most impacted by the 2015 T2 HCC policy (Regions 3, 6, 10, and 11) were compared with those in the remaining regions with respect to LRT practices during waitlisting, and tumor characteristics pre-LT and on explant. These UNOS Regions were selected as the absolute change in median waiting time prepolicy versus postpolicy exceeded 120 d.

### Statistical Analyses

Descriptive statistics were used to compare demographic and clinical parameters, tumor characteristics on submitted applications, and

As an exploratory analysis, the effect of the 2015 HCC policy on posttransplant HCC recurrence was investigated. To assess this endpoint, data from the UNOS posttransplant malignancy file were used. Unadjusted time-to-event analyses were performed using Kaplan-Meier curves. Patients were censored at time of first documented HCC recurrence or date of last follow-up in those without identified recurrence. The prepolicy and postpolicy cohorts were compared using the log-rank test. As a sensitivity analysis, the endpoint of interest additionally included deaths attributed to recurrent HCC in those without documented recurrent HCC.^23^ These subjects were censored at date of death.

All analyses were performed using STATA version 16 (College Station, TX). This study was approved by the Institutional Review Board at the University of Pennsylvania.

## RESULTS

The primary analytic cohorts included 6562 prepolicy candidates waitlisted with a first T2 HCC exception application between Janaury 1, 2010 and December 31, 2014, and 2345 postpolicy candidates waitlisted with a first application between October 8, 2015, and February 12, 2019. As aforementioned, these cohorts were restricted to patients with active T2 HCC exception points at the time of LT, waitlist removal for other reasons or end of follow-up.

At initial transplant listing, the postpolicy cohort was significantly older, less likely to have hepatitis C, and more likely to have cirrhosis due to nonalcoholic steatohepatitis (NASH) or alcohol use (Table 1). There was no difference in the proportion applying for exception points within 30 d of waitlisting between the 2 groups (*P* = 0.7). At the time of first exception application, postpolicy candidates had fewer tumors and lower AFP levels (*P* < 0.001 for both). Though statistically significant, largest tumor size was not clinically different (median 2.4 cm [interquartile range (IQR), 2.1–3.0] versus 2.4 cm [IQR, 2.1–2.9]; *P* < 0.001). Preexception application LRT practices were not significantly different before and after implementation of the 2015 HCC policy (Table 1).

**Table 1.**
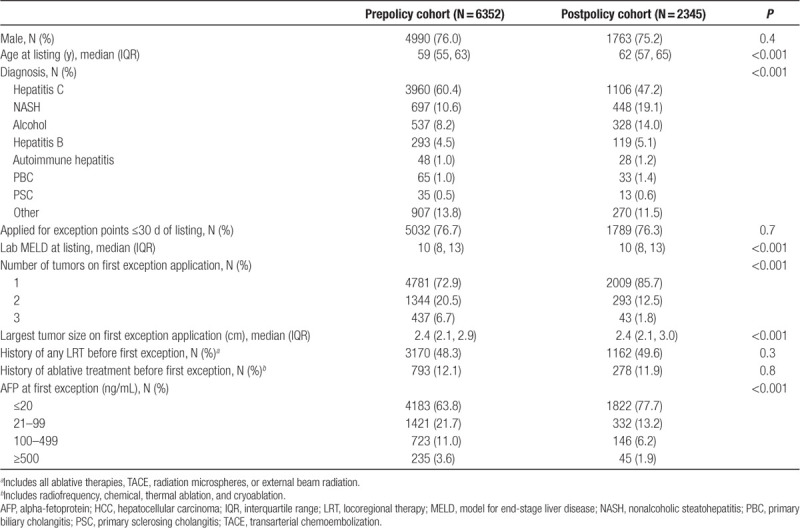
Demographic, clinical, and tumor characteristics of patients with T2 HCC exception points in the prepolicy and postpolicy eras

**FIGURE 1.**
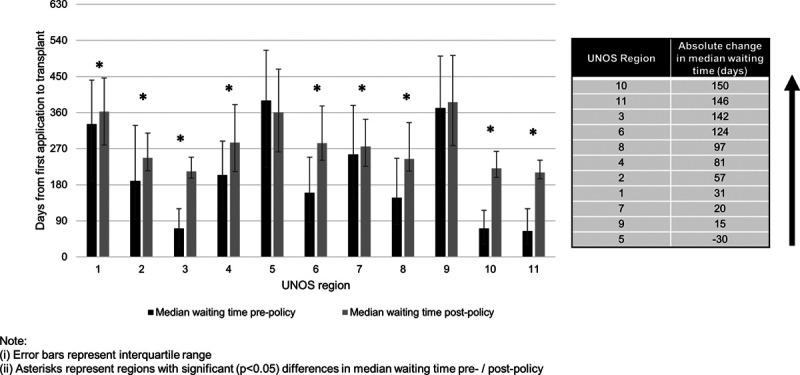
Changes in waiting time from first application date to liver transplantation by UNOS Region (N = 7567). UNOS, United Network for Organ Sharing.

Overall, 5844 prepolicy and 1723 postpolicy candidates were transplanted. Nationally, overall median time from initial exception application to LT was significantly longer after implementation of HCC exception policy (169 d [IQR, 77–327] prepolicy versus 259 d [IQR, 211–361] postpolicy; *P* < 0.001). In most regions, differences were small, and in UNOS Regions 5 and 9, these differences did not reach statistical significance (Figure 1). However, marked increases in waiting time were observed in UNOS Regions 3, 6, 10, and 11, where prewaiting/postwaiting time differences exceeded 120 d.

**FIGURE 2.**
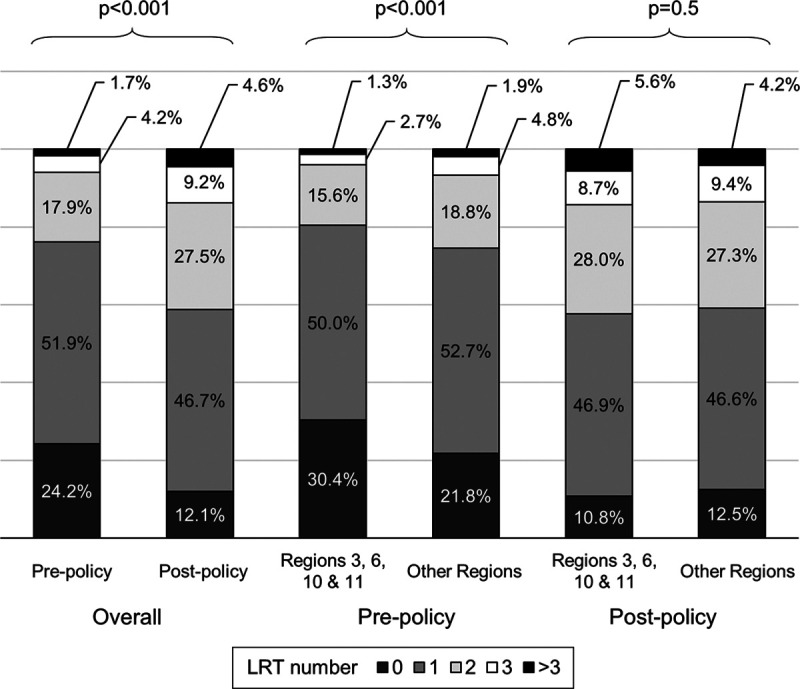
Number of LRTs administered during waitlisting before and after the 2015 HCC policy change overall, and comparing Regions 3, 6, 10, and 11 (previously short-wait regions) to other UNOS Regions. LRT, locoregional therapy; UNOS, United Network for Organ Sharing.

Postpolicy patients underwent more frequent LRT than the prepolicy cohort (*P* < 0.001; Figure 2). Postpolicy LT recipients had significantly fewer tumors reported on their last application (*P* < 0.001), and smaller median size of their largest tumor (*P* < 0.001; Table 2). In fact, 58.1% of recipients in the postpolicy era had a residual tumor size of 0 cm on their last application (ie, complete radiologic response), compared with 36% in the prepolicy era (*P* < 0.001). The proportion of recipients with elevated AFP was also significantly lower in the postpolicy era (*P* < 0.001; Table 2).

**Table 2.**
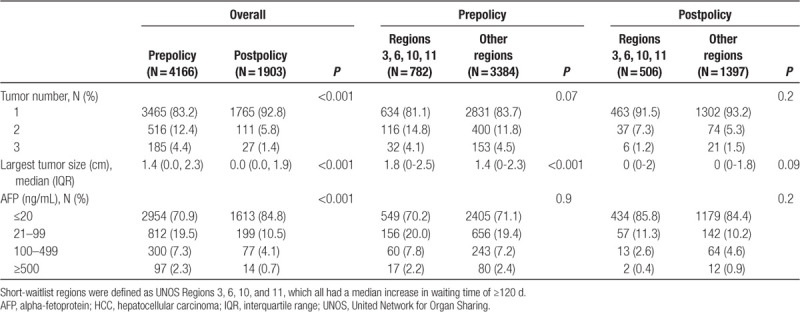
Tumor characteristics on last application among candidates with >1 exception application before and after the 2015 HCC policy change overall, and comparing Regions 3, 6, 10, and 11 (previously short-wait regions) to other UNOS Regions

Last imaging tumor characteristics were subsequently compared between UNOS Regions 3, 6, 10, and 11 (increase in waiting time >120 d as a result of the 2015 T2 HCC policy change) and the remaining 7 UNOS Regions (Table 2). Before the policy change, candidates in these short-wait regions underwent significantly less LRT (*P* < 0.001), but geographic differences were no longer observed postpolicy (*P* = 0.5; Figure 2). This change was largely driven by more aggressive LRT UNOS Regions 3, 6, 10, and 11. Significant regional differences in largest pre-LT tumor size were observed prepolicy (median 1.8 cm [IQR, 0–2.5] for Regions 3, 6, 10, and 11 versus 1.4 cm [IQR, 0–2.3] in other regions; *P* < 0.001), and were also no longer identified postpolicy (median 0 cm in all regions, *P* = 0.09; Table 2). Of note, there was no geographic variability in the distribution of AFP levels at the time of last exception application prepolicy or postpolicy (*P* = 0.9 and *P* = 0.2, respectively).

On explant, there was no difference in the total number of tumors identified between the 2 cohorts overall (*P* = 0.6; Table 3). There was no change in the size of largest tumor found on explant (median 2.5 cm [IQR, 1.8–3.5] prepolicy versus 2.4 [IQR, 1.6–3.6] postpolicy; *P* = 0.9). The postpolicy cohort had a slightly lower prevalence of tumor(s) with microvascular or macrovascular invasion (12% versus 14.6%; *P* = 0.02). They were also less likely to have ≥1 tumor with no necrosis (32.55% versus 38.48%; *P* < 0.001), and more likely to have ≥1 tumor with complete necrosis (23.9% versus 18.4%; *P* < 0.001). Of the 1739 patients with ≥1 nonnecrotic tumor, the median explant tumor size of nonnecrotic lesions was 1.4 cm (IQR, 0.9–2 cm). In only 35.5% of recipients, the number of nonnecrotic tumors on explant exceeded to the number of lesions known at the time of last pre-LT imaging, suggesting that in the majority of cases, these tumors were not incidental. The proportion of incidental nonnecrotic tumors on explant did not differ by policy era (*P* = 0.4).

**Table 3.**
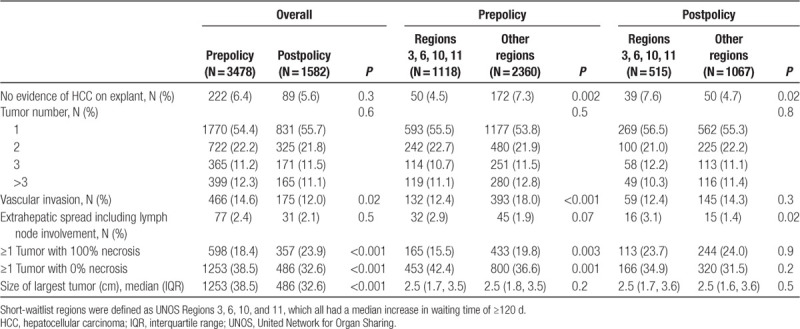
Explant findings before and after the 2015 HCC policy change overall, and comparing Regions 3, 6, 10, and 11 (previously short-wait regions) to other UNOS Regions

Explant findings prepolicy versus postpolicy were also compared between UNOS Regions 3, 6, 10, and 11 and the remaining 7 UNOS Regions (Table 3). Interestingly, there has been a shift toward more frequent false-positive HCCs in previously short-wait regions (4.5% prepolicy versus 7.6% postpolicy; *P* = 0.01), whereas the opposite was seen in other UNOS Regions (7.3% versus 4.7%; *P* = 0.004). No significant regional differences in tumor number or size on explant were noted (Table 3). Vascular invasion was more prevalent in medium- and long-wait time regions prepolicy (18% versus 12.4% Regions 3, 6, 9, and 10; *P* < 0.001), and no longer so postpolicy (14.3% versus 12.4%; *P* = 0.3). Extrahepatic HCC was more prevalent in previously short-wait regions postpolicy (3.1% versus 1.4%; *P* = 0.02). Whereas prepolicy patients in Regions 3, 6, 10, and 11 were less likely to be transplanted with complete pathologic response of ≥1 tumor (15.5% versus 19.8% all other regions; *P* = 0.003), postpolicy no regional differences were noted (23.7% versus 24%; *P* = 0.9). The inverse trend was noted for explants with at least 1 fully untreated tumor, which was more frequent in previously short-wait regions prepolicy (42.4% versus 36.6%; *P* = 0.001) and no longer so postpolicy (34.9% versus 31.5%; *P* = 0.2).

In all patients, the mean difference in largest tumor size between last reported imaging and explant pathology was significantly greater in the postpolicy era: +1.71 cm (±2.17 cm) versus +0.6 cm (±1.71 cm) in the prepolicy era (*P* < 0.001). This difference was greater among recipients in Regions 3, 6, 10, and 11, for whom the mean imaging-explant size discrepancy was +1.81 (±4.94 cm) postpolicy versus +0.66 cm (±1.59 cm) prepolicy (*P* < 0.001). The mean difference in largest tumor size was less pronounced among recipients in other UNOS Regions: mean +1.76 (± 2.18 cm) postpolicy versus +1.2 cm (±1.88) prepolicy (*P* < 0.001). The proportion of explants with a greater number of tumors on explant than that reported on the last application was not statistically different prepolicy versus postpolicy overall (38.6% and 41.2%, respectively). However, prepolicy, recipients in previously short-wait regions were significantly less likely to have a greater number of tumors on explant than on last reported imaging (35.5% versus 40.2%, respectively; *P* = 0.01). Postpolicy, no regional differences were noted: 40.3% in UNOS Regions 3, 6, 10, and 11 versus 41.6% in all other regions (*P* = 0.6). In the postpolicy cohort, there was a greater proportion of explants with HCC measured as outside of Milan criteria (ie, 1 tumor >5 cm or 2–3 tumors >3 cm or >3 tumors; 22.1 versus 18.3%; *P* = 0.002). However, it should also be noted that there was no difference in the proportion of recipients with >3 tumors (ie, who exceeded Milan criteria on the basis of tumor number alone), according to policy era (11.5% prepolicy versus 10.4% postpolicy; *P* = 0.3).

In the exploratory analysis, 259 (4.7%) post-LT recurrences were observed in the prepolicy cohort and 56 (2.1%) in the postpolicy cohort. Though recurrence rates were significantly different between groups (*P* < 0.001), follow-up time was markedly longer in the prepolicy cohort (median 5.3 y versus 1 y, respectively), as anticipated. The distribution of and time to first HCC recurrence was not significantly different prepolicy and postpolicy (*P* = 0.7; Figure S1, SDC, http://links.lww.com/TXD/A271). Similar results were obtained in the sensitivity analysis including deaths attributed to HCC recurrence in the endpoint definition (data not shown).

## DISCUSSION

The 2015 T2 HCC exception policy led to increased waiting time for the majority of candidates, resulting in a more aggressive use of evidence-based therapies before transplant. As a result, LT recipients since October 2015 demonstrate overall more favorable tumor characteristics both while on the waitlist and on liver explant. Our study additionally builds on recent research suggesting that the 2015 policy has led to greater geographic equity in transplant access for HCC candidates nationally: we show that the downstream effect of reduced variability in waiting time is that of greater homogeneity in treatment practices and residual tumor burden at the time of LT, and thus the potential for more widespread improvements in HCC recurrence in the future.

As a result of more aggressive LRT in short-wait regions, pre-LT geographic differences in rates of tumors with complete pathologic response or fully untreated tumors on explant were no longer observed after October 2015, a positive consequence of the policy. In contrast, rates of vascular invasion and extrahepatic spread decreased in medium- and long-wait regions postpolicy but were grossly unchanged in UNOS Regions 3, 6, 10, and 11. These observations could be due to more frequent waitlist dropout of patients with more advanced HCC in medium- and long-wait regions and more selective waitlist practices for borderline patients in these areas given longer anticipated waiting time.

The 2015 T2 HCC policy was also found to have other unintended effects on recipients in short-wait regions. For uncertain reasons, UNOS Regions 3, 6, 10, and 11 experienced an increase in the prevalence of false-positive HCC. It is possible that centers in these areas adapted to the 6-mo waiting rule with more aggressive interpretation of imaging criteria, perhaps under the assumption that borderline lesions would evolve into definitive HCC during waitlisting. However, we also observed that recipients in these areas were more likely to have a greater number of tumors on explant than on last reported imaging in the postpolicy era, which may refute this hypothesis. Other investigators have also demonstrated that significant center- and regional-level variation exists in the potential for understaging HCC before LT.^24^

Greater discrepancies in largest tumor size between last imaging and explant were noted in this study for recipients in the postpolicy era, which is likely the result of more aggressive LRT. Indeed, the more frequent use of these therapies has possibly rendered the ability to accurately predict residual tumor viability on imaging more challenging and has yielded larger treatment zones that extend beyond the “true” tumor borders on explant pathology review. This is supported by the greater heterogeneity in imaging-explant tumor size differences observed in the postpolicy era, and likely also explains the increased prevalence of explants beyond Milan criteria. Unfortunately, UNOS explant reporting does not distinguish the sizes of viable versus nonviable portions for treated lesions, which is an inherent limitation. Although tumor growth could have occurred between last imaging and LT, this would be expected to be nondifferential by policy era given the need for timely surveillance imaging while listed with exception points.

Significant differences in the characteristics at initial application were also noted in the prepolicy versus postpolicy era. The increasing age of patients in this study, as well as the shifts in liver disease pathogenesis, are primarily a reflection of overall temporal trends in the characteristics of waitlisted patients in the United States.^25^ Increasing age is an independent risk factor for tumor understaging and thus may have contributed to our findings.^24^ In contrast, changes in the prevalence of LTs for NASH and hepatitis C virus would be expected to reduce the risk of post-LT HCC recurrence over time.^26,27^ Moreover, temporal shifts in the selection of HCC candidates for LT waitlisting in the setting of elevated AFP may reflect the increasing awareness of AFP as an important predictor of adverse post-LT outcomes over time, rather than being due to the policy change alone.^21,28,29^ The observed decrease in the waitlisting of candidates with multiple tumors may also relate to this, or could be explained by the prolonged waiting time anticipated as a result of the 2015 T2 policy change.

Differences in baseline tumor biology and waitlist drop out of patients with more aggressive tumors likely contributed to the increased prevalence of favorable tumor characteristics on explant in the postpolicy era. Moreover, the restriction of T2 exception points to candidates with AFP ≤1000 ng/mL in 2017 likely further contributed to the dropout of patients with unfavorable HCCs. Given the practice shifts toward transplantation of patients with more favorable tumor characteristics, it is likely that the survival benefit of LT derived by T2 HCC exception candidates has changed over time.^30^ Additional research will need to determine whether the current national trend toward more aggressive LRT will lead to significant improvements in post-LT outcomes for recipients with HCC at large, particularly in the context of the changing demographics of HCC recipients over time. Our exploratory analysis suggests that there has not been a significant change in post-LT HCC recurrence since 2015.

Our study expands several recent studies evaluating the impacts of the 2015 HCC exception policy change.^13–15^ Ishaque et al showed that the policy resulted in decreased transplantation rates and increased mortality/dropout for HCC candidates, attenuating the advantage they have compared with non-HCC candidates. Brondfield et al demonstrated longer waitlist times, particularly in short-wait regions, and decreased regional variability in dropout rates. Finally, Nagai et al showed no change in posttransplant mortality or proportion of patients within Milan criteria on explant. By comprehensively evaluating LRT practices and explant findings prepolicy and postpolicy, our findings provide a mechanism for the changes in waitlist and post-LT patient outcomes observed by these other investigators.

There were several limitations to our study. Exception applications cannot fully capture a patient’s story with the same granularity of data as physician chart documentation and radiology reports. Additionally, post-LRT tumor sizes on both imaging and pathology reports are likely to be subject to intrarater variability, both among readers and transplant centers. These inconsistencies may have been affected by the greater use of LRT in the postpolicy era. This study was also limited by the incomplete availability of pathology data, which are only available for LTs performed on or after April 8, 2012. Finally, we limited the study population to those with active T2 exception points at the time of waitlist removal or end of follow-up. This ensured that all imaging findings were available during candidates’ waiting time but also led to a smaller-sized postpolicy cohort than that reported by other investigators.^13–15^

Since the 2015 T2 HCC policy change evaluated in this study, UNOS now limits the maximum exception score to each center’s median MELD at transplant minus 3 (MMaT-3). Candidates are still required to wait a mandatory 6 mo before receiving exception points. The MMaT-3 policy was instituted with a similar goal of reducing the inequity in LT access between HCC and non-HCC patients, and, as a consequence, additionally prolongs waitlist times for HCC patients in many areas of the United States. Moreover, a new organ distribution system was implemented in February 2020 based on acuity circles that is likely to further impact the access to LT for T2 HCC recipients. According to data from UNOS, the number of LTs for T2 HCC has decreased by approximately one-third between 2018 and 2019. Perhaps most impressive, between January 1, 2020, and April 30, 2020, only 87 transplants have been performed nationally for HCC. By comparison, 1236 LTs for T2 HCC were performed in all of 2019.^31^ Given these changes, it is likely that patients with T2 HCC will require more LRT during waitlisting to prevent tumor progression and waitlist dropout. In some cases, this will further allow patients to achieve complete pathologic response before LT. However, there is also a risk that a greater proportion of patients over time will be transplanted with partially treated or new untreated tumors due to issues with LRT tolerability or hepatic decompensation from treatment, potentially impacting their risk of recurrent HCC post-LT. From a geographical standpoint, it is also likely that these recent policies will lead to further homogeneity in the management of T2 HCC nationally.

In conclusion, our retrospective cohort study of the UNOS database demonstrated increased adoption of pretransplant LRT with more favorable explant findings among T2 HCC candidates after the 2015 policy change. Moreover, as waiting time has become more homogeneous across the country, geographic variability in pre-LT treatment practices and explant findings has lessened. Additional research will need to explore the impacts of increased LRT on long-term posttransplant outcomes, as well as of the 2019 MMaT-3 policy on geographic differences in tumor characteristics at LT.

## ACKNOWLEDGMENTS

This work was supported in part by Health Resources and Services Administration contract 234-2005-370011C. The content is the responsibility of the authors alone and does not necessarily reflect the views or policies of the Department of Health and Human Services, nor does mention of trade names, commercial products, or organizations imply endorsement by the U.S. Government.

## Supplementary Material


