# Supplementary figures and images for: T2 Hepatocellular Carcinoma Exception Policies That Prolong Waiting Time Improve the Use of Evidence-based Treatment Practices
Source: Transplant Direct. 2020 Aug 21;6(9):e597. doi: 10.1097/TXD.0000000000001039 (PMC7447448; doi:10.1097/TXD.0000000000001039)

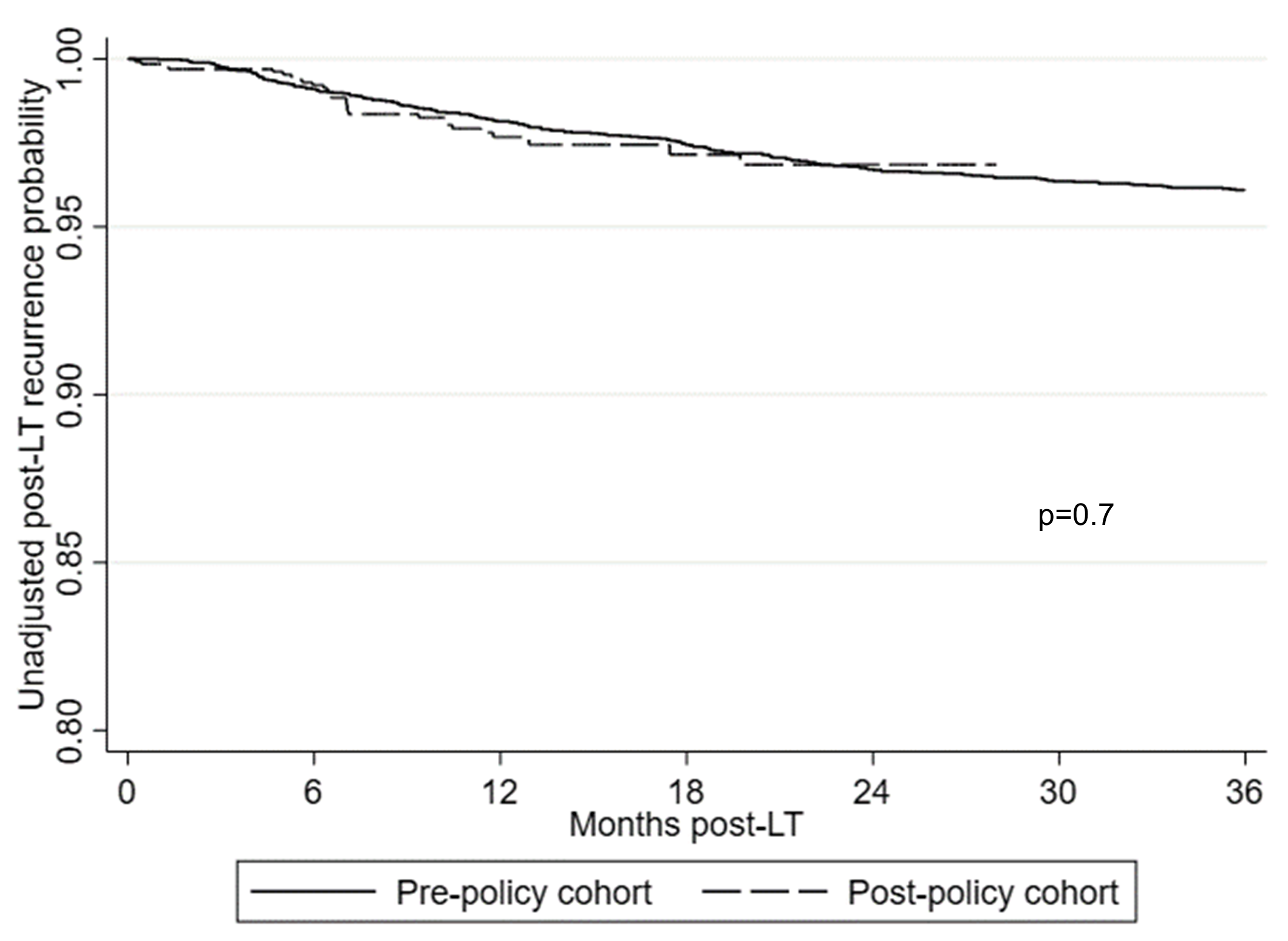

Supplement: Supplementary file 1 [file txd-6-e597-s001.tif]
